# A new multiple robot path planning algorithm: dynamic distributed particle swarm optimization

**DOI:** 10.1186/s40638-017-0062-6

**Published:** 2017-11-02

**Authors:** Asma Ayari, Sadok Bouamama

**Affiliations:** Cosmos Lab, ENSI, University of Manouba, 2010 Manouba, Tunisia

**Keywords:** Multiple robots, Path planning, Stagnation, Local optimum detectors

## Abstract

Multiple robot systems have become a major study concern in the field of robotic research. Their control becomes unreliable and even infeasible if the number of robots increases. In this paper, a new dynamic distributed particle swarm optimization (D^2^PSO) algorithm is proposed for trajectory path planning of multiple robots in order to find collision-free optimal path for each robot in the environment. The proposed approach consists in calculating two local optima detectors, LOD_pBest_ and LOD_gBest_. Particles which are unable to improve their personal best and global best for predefined number of successive iterations would be replaced with restructured ones. Stagnation and local optima problems would be avoided by adding diversity to the population, without losing the fast convergence characteristic of PSO. Experiments with multiple robots are provided and proved effectiveness of such approach compared with the distributed PSO.

## Background

The concept of multiple robot systems (MRS) began in the 1990s, in particular in works regrouping mobile robots, gathering objects [1] and robot colonies [2, 3]. Arai et al. [4] identified seven primary research themes in the MRS: biological inspirations, communication, architectures, location/cartography/exploration, transport and handling of objects, motion coordination and reconfigurable robots.

Multiple robot systems are well known by the synchronization process and having better spatial distribution capability as compared to a single robot. This coordination addresses the problem of how teams of autonomous mobile robots can share the same workspace while avoiding interference with each other, collision with static obstacles and/or while achieving group motion objectives.

There are two basic approaches to solve the problem of multiple robot path planning: centralized and distributed. In the case of the centralized approach, each robot is treated as a composite system, and planning is done in a composite configuration space, formed by combining the configuration spaces of the individual robots. While in the case of the distributed approach, paths are first generated for robots independently and then their interactions are considered. The advantage of centralized approaches is that they always find a solution when there is one. However, the practical difficulty is the temporal complexity which is exponential in composite configuration space. Distributed planners help generate robot trajectories independently before using different strategies to resolve potential conflicts. But, they are incomplete in nature (probabilities of various and varied configurations) and can therefore lead to blocking situations. This distributed approach can be applied to each robot taking into account the positions and orientations of all other robots at each point in time. Thus, the general problem is reduced to several versions of the planning problem for a single mobile robot in the presence of other robots which move in the presence of fixed obstacles. A trajectory of the robot is then found by the search of a path from the start to the arrival thanks to the spatial–temporal configuration.

The control of the MRS becomes unreliable and even infeasible if the number of robots increases. In addition, the multiple robot path planning problem becomes more and more complex. The latter has been extensively studied since the 1980s. Swarm behavior has proven its effectiveness in such problems thanks to interesting properties like robustness, flexibility and scalability. One of the successful optimization methods is particle swarm optimization (PSO) algorithm.

This paper proposed a novel approach to determine the optimal trajectory of the path for distributed multiple robots system using dynamic distributed particle swarm optimization (D^2^PSO), where each robot is considered to be a mobile, autonomous and physically independent agent.

The remaining part of the paper is outlined as follows. “Literature survey of particle swarm optimization use in MRS path planning” section covers briefly the latest works done in the MRS path planning search domain using different PSO variants. Formulation of the problem for multiple robot path planning has been elaborated in “Problem formulation for multiple robot navigation” section. “Obstacle avoidance approach” section describes our obstacle avoidance approach. The classical particle swarm optimization, dynamic distributed double guided particle swarm optimization algorithm and dynamic distributed particle swarm optimization are described in the “Particle swarm optimization (PSO) for MRS path planning” section. “Conclusion” section demonstrates the computer simulation for path planning of multiple robots.

## Literature survey of particle swarm optimization use in MRS path planning

Since the inception of PSO [5, 6], several variants have been proposed to improve the performance of original PSO. The first versions of PSO for MRS were proposed in [7–9] to find a target in a given environment, and studies have demonstrated that the PSO algorithm has acceptable performances in the searching task. In the study of Chakraborty et al. [10], behavioral cooperation of the robots was realized through selection of alternative local trajectories for collision avoidance among teammates. In fact, he compared the performances using differential evolution (DE) with a PSO-based realization.

The authors present in [11] PSO-based technique for determining the optimal set of parameters for a second PSO for collective robotic search. Particle swarm optimization technique was used to optimize the velocity parameters of robots in [9], to arrive at the shortest collision-free trajectory, satisfying dynamic constraints. A hybrid technique for the control of swarms of robots, based on particle swarm optimization (PSO) and consensus algorithms, is presented in [12]. A MOPSO algorithm is utilized in [13] to generate trajectories for mobile robots that are working on the environments that the robots are working on and may be included danger sources. Darvishzadeh and Bhanu [14] present a framework to use a modified PSO (MPSO) algorithm in a multiple robot system for search task in real-world environments. Nakisa et al. [15] also proposes a new method (APSO) to create an efficient balance between exploration and exploitation by hybridizing basic PSO algorithm with A-star algorithm. Nakisa proposes a method based on the multi-swarm particle swarm optimization (PSO) with local search on the multiple robot search system to find a given target in a complex environment that contains static obstacles [16]. Rastgoo et al. [17] proposed an algorithm named the “modified PSO with local search (ML-PSO)” applied in the exploration search space by adding a local search algorithm such as A-star to guarantee global convergence with a reduction in the search time. Allawi and Abdalla [18] used PSO combined with reciprocal velocity obstacles (RVO) method, in order to choose the best paths for robots without collision and to get to their goals faster. Das [19] proposed a new methodology to determine the optimal trajectory of the path for multiple robot in a clutter environment using hybridization of improved particle swarm optimization (IPSO) with an improved gravitational search algorithm (IGSA).

A hybridization of improved particle swarm optimization (IPSO) with differentially perturbed velocity (DV) algorithm (IPSO-DV) was also proposed by Das et al. [20] for trajectory path planning of multiple robots in a static environment. Abbas et al. discusses in [21] an optimal path planning algorithm based on an adaptive multi-objective particle swarm optimization algorithm (AMOPSO) for five robots to reach the shortest path. The algorithm PSO-NAV presented in Raffaele Grandi’s work [22] focuses on the possibility to drive a group of very simple robots from a starting zone to a final one inside a maze-like environment unknown a priori.

## Problem formulation for multiple robot navigation

The problem formulation for multiple robot path planning is provided in this section. We consider a group of mobile robots to navigate by maintaining predefined geometric shapes (line, column, triangle, etc.), controlling the location of each robot relative to the others. The geometric formation is established from predetermined initial positions, or even from random positions, and is maintained during the movement of the group. This navigation must ensure the avoidance of obstacles in the environment. This kind of navigation is useful in many cooperation tasks such as moving a sports field, transporting or manipulating objects involving several mobile robots.

Multiple robot path planning problem is formulated by considering the set of principles using the following assumptions:

1. For each robot, the current position (recent position) and goal position (target position) is known in a given reference coordinate system.
2. Each robot is performing its action in steps until all robots reached in their respective target positions.

The following principles have been taken care of for satisfying the given assumptions.

1. For determining the next position from its current position, the robot tries to align its heading direction toward the goal.
2. The alignment may cause a collision with the robots/obstacles (which are static in nature) in the environment. Hence, the robot has to turn its heading direction left or right by a prescribed angle to determine its next position.
3. If a robot can align itself with a goal without collision, then it will move to that position.
4. If the heading direction is rotated to the left or right, then it is required for the robot to rotate the same angle about its *z*-axis.

## Obstacle avoidance approach

Plenty of algorithms for obstacle avoidance were mentioned in the robotic literature [23–25].

The obstacle avoidance approaches in MRS studies aim to find a path from an initial position *S* of a robot to a desired goal position *G*, with respect to positions and shapes of known obstacles *O*. The penalty function to be minimized by the planning algorithm consists of two parts. While the first one evaluates a length of the trajectory (or time needed to execute the trajectory), the second part ensures safety of the path (i.e., distance to obstacles).

**Fig. 1:**
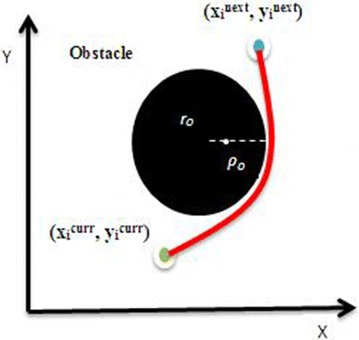
Selection of next position (*x*_*i*_^next^, *y*_*i*_^next^) of robot *i* from current position (*x*_*i*_^curr^, *y*_*i*_^curr^) for avoiding collision with obstacle (*r*_*o*_, *ρ*_*O*_)

To solve the latter problem, we propose a method which is able to detect collisions between the robot and an object (figure 1). Let us define a repulsive function $F_{o}$ in a region $Z$ around an obstacle $O$. The region $Z$ is defined as a circular disk centered at $r_{o}$ with radius $\rho_{O}$; the parameter $\left( {\rho_{O} + \varepsilon } \right)$ is the minimum distance that the robot should keep with respect to the boundary of the obstacle $O$. $\varepsilon$ represents the minimum allowed distance between the robot and the obstacle. The repulsive function can then be defined out of Eq. (1), where $\left( {x, y} \right)$ is the position of the robot and $\left( {r_{ox} , r_{oy} } \right)$ is the obstacle position.

1 $$F_{o} = \sqrt {\left( {x - r_{ox} } \right)^{2} + \left( {y - r_{oy} } \right)^{2} }$$

Afterward, we define a Boolean function $\delta_{ij}$ described in (2), where *i* refers to the *i*th robot and *j* refers to the *j*th obstacle in the environment.

2 $$\delta_{ij} = \left\{ {\begin{array}{*{20}l} {1,} \hfill & {\text{if}}\; {F_{{o_{ij} }} \le \varepsilon } \hfill \\ 0 \hfill & {\text{Otherwise}} \hfill \\ \end{array} } \right.$$

Robots must be able to handle limited sensing range for the obstacles through considering the latter function value in order to check collision (Fig. 1).

## Particle swarm optimization (PSO) for MRS path planning

### Classic PSO

Particle swarm optimization (PSO) is a stochastic optimization method for nonlinear functions based on the reproduction of social behavior developed by Berhart and Kennedy [5, 6] in 1995.

The origin of this method comes from the observations made during computer simulations on grouped flights of birds and fish [26]. These simulations highlighted the ability of individuals in a moving group to maintain an optimal distance between each other’s and to follow a global movement in relation to neighbors one.

To apply the PSO, we have to define a particle search space and an objective function to optimize. The principle of the algorithm is to move these particles so that they find the optimum.

Each of these particles is provided with:

- A position, that is, its coordinates in the definition set.
- A speed that allows the particle to move. In this way, during the iterations, each particle changes its position. It evolves according to its best neighbor, its best position and its previous position. This evolution makes it possible to fall on an optimal particle.
- A neighborhood, that is, a set of particles that interact directly with the particle, especially the one with the best criteria.

At every moment, each particle knows:

- Its best position visited. The value of the calculated criterion and its coordinates are essentially retained.
- The position of the best neighbor of the swarm that corresponds to the optimal scheduling.
- The value of the objective functions because it is necessary to compare the value of the criterion given by the current particle with the optimal value.

PSO is initialized with a group of random particles (solutions) and then searches for optima by updating generations. In every iteration, each particle is updated by following two “best” values. The first one is the best solution (fitness) the particle has achieved so far. This value is called pBest. Another “best” value that is tracked by the particle swarm optimizer is the best value obtained so far by any particle in the population. This best value is a global best and called gBest.

After finding the two best values, the particle updates its velocity and positions with following equations:

3 $$v_{n + 1} = wv_{n} + c_{1} *{\text{rand}}*({\text{pBest}}_{n} - p_{n} \, ) + c_{2} *{\text{rand}}*({\text{gBest}}_{n} - p_{n} ),$$

4 $$p_{n + 1} = p_{n} + v_{n + 1} .$$

where *w* is the inertia coefficient which slows velocity over time; *v*_*n*_ is the particle velocity; *p*_*n*_ is the current particle position in the search space; pBest_*n*_ and gBest_*n*_ are defined as the “personal” best and global best; rand is a random number between (0, 1); *c*_1_ and *c*_2_ are the acceleration coefficients. The stop condition is usually the maximum number of allowed iterations for PSO to execute or the minimum error requirement. As with the other parameters, the stop condition depends on the problem to be optimized.

### D^3^GPSO: the dynamic distributed double guided particle swarm optimization algorithm

The D^3^GPSO introduced by Bouamama in [4, 27] is a distributed PSO. It is a group of agents dynamically created and cooperating in order to solve a problem. Each agent performs locally its own PSO algorithm.

Inspired by works in [1, 2, 4, 28], this algorithm uses the same principle as the D^3^G^2^A [2], and it consists on dividing the initial population into subpopulations and affecting each one to an agent. Each agent is also called specie agent and is responsible of a set of particles having their fitness values in the same range. This range is called FVR (for fitness value range), and it is the specificity of the specie agent Specie_FVR_. The species agents cooperate all by exchanging solutions to reach the optimal one. In fact, each one executes its own double guided PSO algorithm. The latter is double guided by the concept of template and the min-conflict heuristic. It is enhanced by new parameters: guidance probability *P*_guid_; a local optimum detector LOD and a weight *ε* (used by species agents to calculate their own PSO parameters).

For the D^3^GPSO, we distinguish also a mediator agent to manage the communication between the species agents. This agent, called interface agent, can also create new species agents if necessary.

The local optimum detector LOD is an operator that we use in the PSO process. It represents the number of iterations in which the neighboring does not give improvement. If the best solution found by a specie agent remains unchanged for LOD generations, we can conclude that the particles are blocked in a local optimum. So, the best particle having this fitness value will be penalized. This variable is given by the user at the beginning of the optimization process, but it is changed by each specie agent according to the attained fitness value.

### D^2^PSO: dynamic distributed PSO for MRS path planning

In 2006, Hereford [9] has introduced a version of the PSO that “distributes” the motion processing among several, simple, compact, mobile robots, called distributed PSO (dPSO). Calculations were done “locally,” that is on each local robot. Simulation results showed that the dPSO appears to be a very good way of coordinating simple robots for a searching task operation. One of the most important advantages was that the algorithm appears to be scalable to large numbers of robots since the communication requirements do not increase as the number of robots is increased.

Although swarm intelligence approaches are attractive methods for robotic target searching problems, these strategies have two important disadvantages: First, they may get stuck on local optima. Second, they have slow progress in terms of fitness function in some situations (slow speed to converge to the target locations).

Inspiring from the D^3^GPSO described in [4, 27], we introduce two new parameters to the PSO: local optima detector for global best LOD_gBest_ and local optima detector for personal best LOD_pBest_. The purpose of the latest parameter is to count the number of successive iterations for which personal best and global best do not give improvement. Since these particles are unable to improve their pBest, they are no more contributing in finding the global optimal solution. This indicates that particles are saturated and require external thrust to boost their power. Dynamic distributed PSO (D^2^PSO) provides thrust by heading particles toward potentially better unexplored regions which also add diversity to the search space. At the same time, when global best gBest is not improving for predefined number of successive iteration, it may be trapped in local optima and mislead other particles by attracting toward it. This also requires some external push that send trapped particle outside local optima position and mitigate its consequences. By this way, the stagnation and local optima problems would be avoided without losing the fast convergence characteristic of PSO since the D^2^PSO would follow the PSO’s behavior for the rest of situations.

**Fig. 2:**
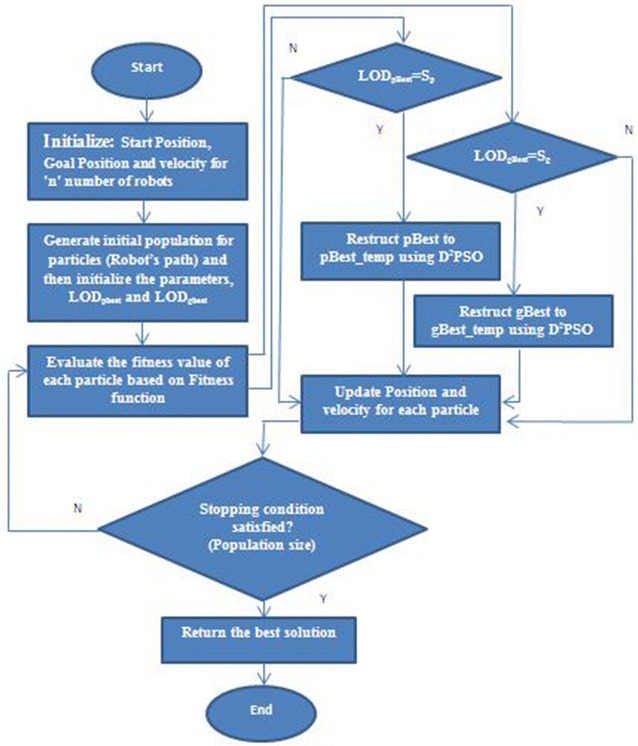
Flow chart of multiple robot path planning using D^2^PSO

The flowchart for multiple robot path planning using D^2^PSO is presented in Fig. 2.

**Fig. 3:**
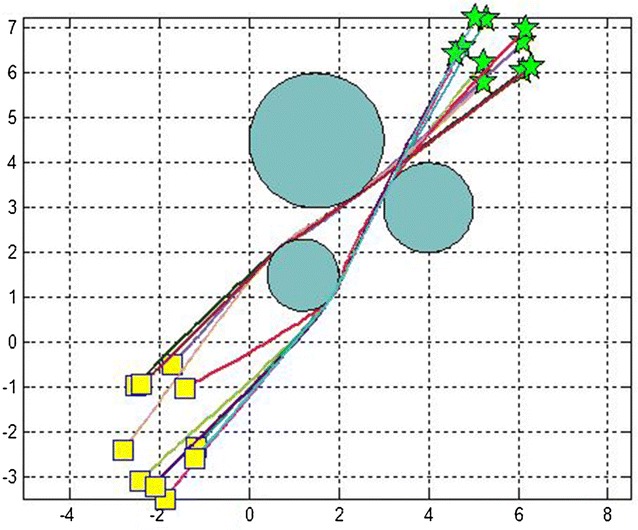
Obstacle avoidance of 10 robots moving in the same environment (rectangles = initial positions, stars = goal positions)

#### Local optimum detector

LOD is a parameter to the whole optimization process, and it will be locally and dynamically updated by each robot [1, 2]. If the personal best of the *i*th particle increases for a specific number of successive generations, we can conclude that the particle optimization sub-process is trapped in a local optimum, and so the LOD_pBest_ will increment, respectively, for gBest and LOD_gBest_.

#### Dynamic concept

Our work consists in implementing a dynamic distributed version of PSO. It is a multi-agent approach. It acquires its dynamic aspect from the agents that it uses. Indeed, they are capable to calculate their own parameters on the basis of the parameters given by the user (*S*_*p*_, *S*_*g*_, *ε*). *ε* ϵ [0, 1].

Particles whose pBest is not improved for a predefined threshold, i.e., LOD_pBest_ = *S*_*p*_, would be restructured as pBest_temp given in (3). Respectively, for gBest, i.e., LOD_gBest_ = *S*_*g*_, would be restructured as pBest_temp given in (4).

5 $${\text{pBest}}\_{\text{temp}}_{i} = {\text{pBest}}_{{i_{1} }} + \frac{{i_{1} }}{{i_{1} + i_{2} }}*({\text{gBest}}\_{\text{hist}}_{{i_{2} }} - {\text{pBest}}_{i} ),$$

6 $${\text{gBest}}\_{\text{temp}}_{i} = \hbox{min} ({\text{pBest}}\_{\text{temp}}_{i} ,{\text{gBest}}_{i} ),$$

where *i*_1_ = random (1, *M*), *M* is the population size, *i*_2_ = random (1, size(gBest_hist), and gBest_hist presents the historical values of gBest.

#### Template concept

The concept of template was introduced by Tsang [29]. In our approach, inspired from D^3^GPSO [27], we will attach particles whose pBest is not improved for successive generations to a template called template_pBest_ and respectively particles whose gBest is not improved for a predefined threshold to a template called template_gBest_. Hence, the new robot path will be influenced by the best reached fitness since each robot would perform its own PSO algorithm guided by template concept [29].

7 $$\alpha = {\text{template}}_{\text{pBest}} /\left( {{\text{template}}_{\text{pBest}} + {\text{template}}_{\text{gBest}} } \right)$$

8 $$\beta = {\text{template}}_{\text{gBest}} /\left( {{\text{template}}_{\text{pBest}} + {\text{template}}_{\text{gBest}} } \right)$$

As mentioned in [27], we define in (8) and (9) the parameters α and β which are function of template_pBest_ and template_gBest_. Their worth is that they articulate the probability for a particle to propagate its knowledge. This confirms the fact that the best particles have more chance to be pursued by others.

## Implementation and experiments

### Experiment configuration

The multiple robot path planning algorithm is implemented in a simulated environment. The simulation is conducted through programming in C language on a Pentium microprocessor, and robot is represented with similar soft-bots of rectangular shape with different path color code. The robot is self-contained in terms of power. It is mobile; it may be limited in terms of steering radius and speed, but it is mobile.

Predefined initial location and goal location for all the robots are assigned. The experiments were conducted with three different radius obstacles and assigned same velocities for each robot at the time of the program run.

The objective function used in the simulation studies was a spherical function given by:

9 $$f = (x_{i} - x_{\text{target}} )^{2} + (y_{i} - y_{\text{target}} )^{2} ,$$

where (*x*_*i*_, *y*_*i*_) is the position of the both and (*x*_target_, *y*_target_) is the position of the target point. The latter is given in Table 1. The spherical function is chosen because it approximates the expected dissipation pattern of chemicals, heat, etc., that would be emitted by real-world targets [9].

**Table 1:** Initial and target points used in simulation

Initial point	*x*	*y*	Target point	*x*	*y*
1	− 2.41	− 3.08	1	5.22	6.22
2	− 1.72	− 0.47	2	6.10	6.70
3	− 1.88	− 3.48	3	4.75	6.59
4	− 2.53	− 0.95	4	6.09	6.05
5	− 1.19	− 2.34	5	5.28	7.18
6	− 2.81	− 2.39	6	5.23	5.78
7	− 1.44	− 1.03	7	6.17	6.99
8	− 2.09	− 3.21	8	4.61	6.41
9	− 2.40	− 0.92	9	6.30	6.13
10	− 0.21	− 2.59	10	5.03	7.21

We have applied the distributed PSO and the proposed D^2^PSO to the same environment. Population size *M* was fixed to 150 and then to 300.

We used the following parameters: inertia weight *w* = 1.0, damping ratio *w*_damp_ = 0.99, personal learning coefficient *c*_1_ = 1.5, global learning coefficient *c*_2_ = 1.5, *S*_*p*_ = 3, *S*_*g*_ = 3.

We did the simulations in an environment with variant number of obstacles. Table 2 gives their positions.

**Table 2:** Description of obstacles present in Fig. 3

Obstacles	Position of obstacles
1	(1, 1.5)
2	(4, 3)
3	(1.5, 4.5)

### Experiment results and analysis

We made several simulation runs. We have run the program for 3, 5, 7 and 10 robots with different initial and target points, for 200 iterations. We evaluated the effectiveness of the algorithm by comparing the path lengths obtained from the basic distributed PSO and the D^2^PSO, ensuring of course the non-collision with the static predefined obstacles (Figure 3).

**Fig. 4:**
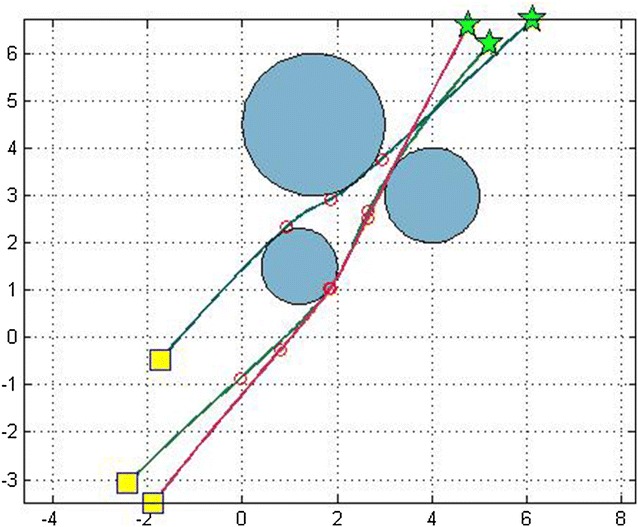
Optimal path of 3 robots using D^2^PSO

**Fig. 5:**
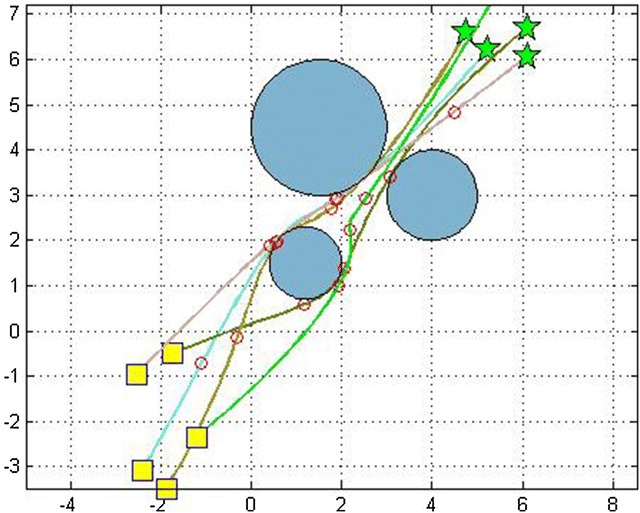
Optimal path of 5 robots using D^2^PSO

The overall effectiveness of the proposed algorithm is shown in Table 3. It confirms that it outperforms the dPSO with respect to the performance metric, i.e., the path length, for different number of robots (figures 4 and 5).

**Table 3:** Path lengths for *M* = 150

Path length *M* = 150		
	Distributed PSO	D^2^PSO
Robot1	12.2489	12.1666
Robot2	11.0501	10.6690
Robot3	12.461	12.1351
Robot4	11.1966	7.8620
Robot5	11.6496	8.1610
Robot6	12.3214	9.1203
Robot7	9.4512	7.6291
Robot8	10.9221	9.7347
Robot9	11.3312	8.5025
Robot10	12.3411	11.7196

Table 4 represents the same previous simulations for *M* = *300* particles. It is clear that we obtain shorter paths for each robot (Figs. 4, 5).

**Table 4:** Path lengths for *M* = 300

Path length *M* = 300		
	Distributed PSO	D^2^PSO
Robot1	12.2421	12.1664
Robot2	11.0449	10.6679
Robot3	12.4518	12.134
Robot4	11.1951	7.8591
Robot5	11.6412	8.1257
Robot6	12.2114	9.1098
Robot7	9.4232	7.6011
Robot8	10.9119	8.6738
Robot9	11.1931	8.4381
Robot10	12.3391	10.6956

**Fig. 6:**
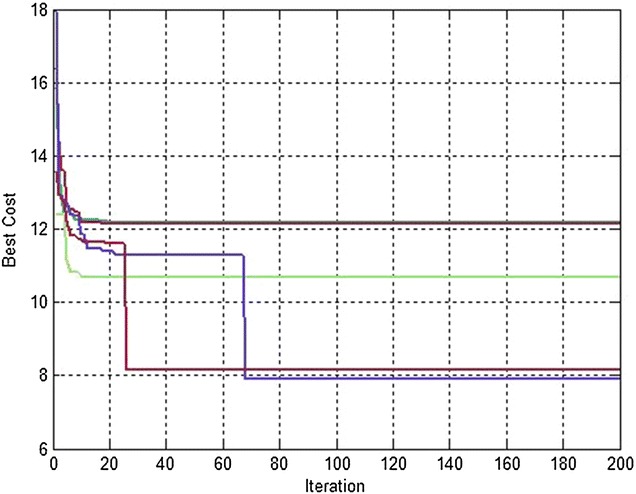
Best cost variation for each robot when applying D^2^PSO (#robots = 5)

**Fig. 7:**
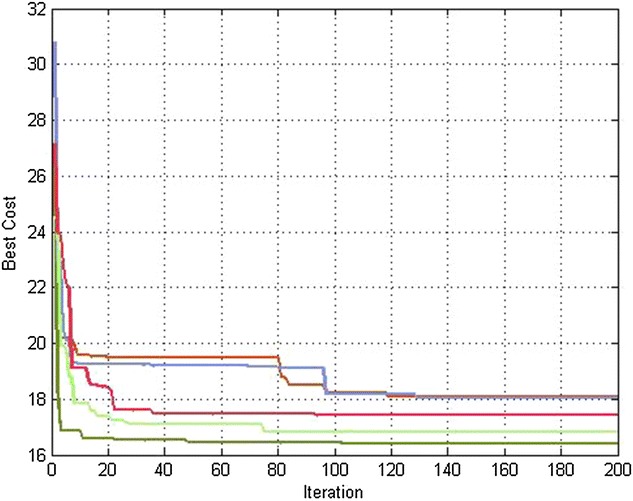
Best cost variation for each robot when applying dPSO (#robots = 5)

Figures 6 and 7 show that the convergence of the objective function to the best value is faster with D^2^PSO approach than dPSO one.

Since restructuring both pBest and gBest depends on the number of robots and the population size, we deduce that each time we increase the number of bots, we obtain better values of the objective function, and respectively when increasing the population size *M*.

**Fig. 8:**
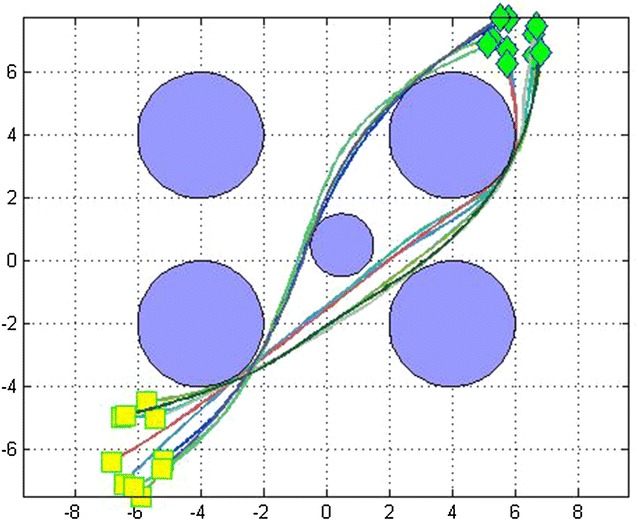
Optimal paths of 7 robots using D^2^PSO with five obstacles

**Fig. 9:**
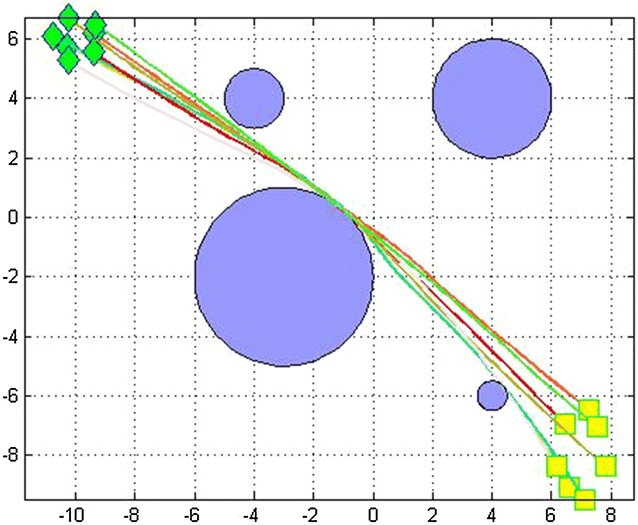
Optimal paths of 7 robots using D^2^PSO with three obstacles

After that, we have added more obstacles and made some changes in initial and target states. We obtained Figs. 8 and 9 for fixed number of robots.

**Fig. 10:**
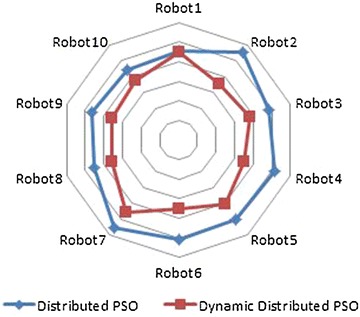
Comparison of time taken to reach best solution in dPSO and D^2^PSO

Moreover, the result of the experiments performed is presented in Table 5 in the term of another performance metric which is the executed time to reach the best solution, i.e., the robot’s shortest path. We run the program with two different values of the population size M (Table 6). This time, the execution time would necessary be bigger as the number of particles increases. Table 5 and Fig. 10 confirm that D^2^PSO outperforms the remaining algorithm with respect to the cited metric for different robots.

**Table 5:** Execution time for *M* = 150

Time (s) *M* = 150		
	Distributed PSO	D^2^PSO
Robot1	40.569334	39.676484
Robot2	48.224949	30.275881
Robot3	43.248937	32.953980
Robot4	45.283051	30.535676
Robot5	45.653371	35.419047
Robot6	48.689387	30.173998
Robot7	49.968918	39.833665
Robot8	40.314716	30.654208
Robot9	40.546997	30.701435
Robot10	40.272204	31.146030

**Table 6:** Execution time for *M* = 300

Time (s) *M* = 300		
	Distributed PSO	D^2^PSO
Robot1	45.103684	44.720177
Robot2	55.595825	35.589437
Robot3	48.550160	37.941275
Robot4	51.812619	35.380362
Robot5	49.960134	40.247571
Robot6	50.862109	35.323138
Robot7	55.566426	45.552622
Robot8	45.396125	35.484535
Robot9	46.271805	35.422918
Robot10	44.100084	37.159384

In addition, we have observed, for each algorithm, the number of iterations each robot takes to find its shortest path. Since the execution time is lower for D^2^PSO than dPSO, number of iterations would consequently be less. Table 7 confirms the latter observation.

**Table 7:** Comparison of number of iterations taken to obtain the shortest path by the different algorithms

# Iterations		
	Distributed PSO	D^2^PSO
Robot1	93	77
Robot2	122	54
Robot3	102	66
Robot4	112	56
Robot5	119	70
Robot6	127	52
Robot7	135	79
Robot8	80	58
Robot9	91	59
Robot10	82	61

## Conclusion

The problem of multiple robot motion planning focuses on computation of paths of different robots such that each robot has an optimal path, and so the overall path of all the robots combined is optimal. Many approaches have been proposed for solving multiple robot path planning problems. Particle swarm optimization algorithm is one of the successful optimization methods in this area. This paper has presented a successful improvement to the PSO algorithm. D^2^PSO ensures diversity to stagnated particles in such a manner that they move to better and unexplored regions of search space. In addition, it does not disturb the fast convergence characteristics of PSO by keeping the basic concept of PSO unaffected. Experimental results show that our approach performs better in escaping local optimum and proves that applying D^2^PSO to multiple robots path planning problem is practical and efficient for large number of robots in environments with variable obstacles.

## Discussion and future work

The main contributions of our research are: (1) finding optimal paths of mobile robots moving together in the same workspace, (2) proposing to use the PSO evolutionary algorithm and (3) ensuring collision-free trajectories.

However, there are still some issues and improvements to be addressed in our future work. First, dynamic obstacles, unknown environment, obstacles’ shapes and collision avoidance should be studied. In this paper, both the environment and obstacles are static relative to the robots, which is applicable in particular cases. In the future, work will be carried out using dynamic obstacles during the multiple robot path planning process. Second, the inter-robot collision should be considered in future experiments. The task planning process for MRS would be also studied in order to ensure best coordination.
